# Safety of Corn and Corn-Based Products Intended for Human Consumption Concerning Fumonisins from a Brazilian Processing Plant

**DOI:** 10.3390/toxins11010033

**Published:** 2019-01-10

**Authors:** Jaqueline Gozzi Bordini, Mario Augusto Ono, Melissa Tiemi Hirozawa, Glauco Tironi Garcia, Edio Vizoni, Elisabete Yurie Sataque Ono

**Affiliations:** 1 Department of Biochemistry and Biotechnology, State University of Londrina, P.O. Box 10.011, Londrina 86057-970, Paraná, Brazil; jgbordini@hotmail.com (J.G.B.); mel_hiro@hotmail.com (M.T.H.); 2 Department of Pathological Sciences, State University of Londrina, P.O. Box 10.011, Londrina 86057-970, Paraná, Brazil; marioono@uel.br; 3 G Tironi Garcia Consultoria Técnica—ME, Andirá 86380-000, Paraná, Brazil; gtironigarcia@gmail.com; 4 Department of Statistics, State University of Londrina, P.O. Box 10.011, Londrina 86057-970, Paraná, Brazil; ediovizoni@bol.com.br

**Keywords:** food safety, daily intake, mycotoxins, cornmeal, endosperm, exposure

## Abstract

Brazil is one of the world’s largest corn producers and is a leader in exportation. Due to intense globalization, corn may be commercialized worldwide and the issue concerning the safety of corn-based products has become a topic of widespread international interest. Dietary exposure evaluation is a relevant criterion for mycotoxin risk assessment. Thus, human exposure to fumonisins were assessed for corn grain and its derivatives (endosperm, cornmeal, and grits; n = 320) sampled from one of the large-scale corn processing plants in Brazil. The total probable daily intake (PDI) for fumonisins in Brazil was 96.9 ng kg^−1^ body weight day^−1^, which corresponds to 5% of the provisional maximum tolerable daily intake (PMTDI) of 2000 ng kg^−1^ b.w. day^−1^ for fumonisins. In countries that import Brazilian corn, the total PDI is lower in European countries (from 35.7 to 177 ng kg^−1^ b.w. day^−1^) and higher in Angola (1553 ng kg^−1^ b.w. day^−1^). Taking into account that dietary exposure in populations in Brazil and importing countries was low, the corn-based products were safe for human consumption regarding fumonisins, even for regions with high corn consumption.

## 1. Introduction

Corn is an economically important crop in Brazil and is a relevant staple food in many developing countries due to its nutritional value [1]. Brazil is the third largest corn producer in the world and produced 97.7 million tons in the 2016/17 crop and exported 30 million tons [2]. Due to intense globalization, corn may be commercialized worldwide and the issue concerning the safety of corn-based products has become a topic of widespread international interest. Tropical and subtropical climates in Brazil favor corn contamination by a variety of mold species, which can deteriorate grain causing loss in hygienic quality, in addition to mycotoxin production [3].

Fumonisins are a group of toxic secondary metabolites produced mainly by fungal species *Fusarium verticillioides* and *F. proliferatum*, primary corn pathogens that cause disease in all the development stages of the plant and are associated to several toxic effects in humans [4,5,6] and animals [7,8]. A total of 28 fumonisin analogues have been isolated and characterized since 1988, but fumonisins B_1_ (FB_1_) and B_2_ (FB_2_) occur at a higher frequency in corn [3].

Fumonisins are associated with neural tube defects [5], esophageal and liver cancer in humans [4,6], and are classified by the International Agency for Research on Cancer—IARC [9] in group 2B (possibly carcinogenic to humans). Due to their health risk, a provisional maximum tolerable daily intake (PMTDI) for fumonisins was estimated at 2 μg kg^−1^ b.w. day^−1^ (2000 ng kg^−1^ b.w. day^−1^) based on the No Observed Adverse Effect Level (NOAEL) of 200 μg kg^−1^ b.w. day^−1^ for renal toxicity and an uncertainty factor of 100 was applied [10].

Brazilian corn dry-milling processing produces the corn derivatives destined for human local and export consumption. It includes the corn endosperm used for breakfast cereal production, or it can be converted to cornmeal and grits and used for cornflakes, snacks, and beer production, respectively. However, fumonisins are thermostable compounds that are not destroyed during this process and they can be detected in corn products constituting a potential hazard to human health [11].

Corn has been considered the cereal that most contributes to total fumonisin intake [12]. Dietary intake of fumonisins can be estimated by the exposure assessment tool, a step of the risk assessment and an essential parameter for quantifying the risk [13]. Evaluation of the exposure degree is performed by comparing the total probable daily intake (PDI) to PMTDI, and the PDI is calculated using the data of food intake and naturally occurring levels of a mycotoxin [14,15,16].

Previous studies have reported the human exposure assessment of fumonisins through corn and corn-based product consumption [17,18,19], but in most of the studies samples were collected from local markets and the fumonisin exposure was evaluated in a specific population group. To the best of our knowledge, there is no information about the safety of corn products processed by Brazilian industry and destined for exportation based on samples obtained from a continuous flow process. Therefore, the objective of the present study was to evaluate the safety of corn and corn-based products (endosperm, cornmeal, and grits) intended for human consumption for fumonisins in Brazil and in countries that import Brazilian corn (Europe, Malaysia, and Angola).

## 2. Results and Discussion

Water activity (a_w_) is an important factor influencing food quality and safety because it is directly related to fungal growth and the extent of fumonisin production [20,21]. Water activity measures the water availability in foods, i.e., the relationship between moisture in foods and the capability of microorganisms to survive on them [22]. Therefore, a_w_ is of significance for the metabolic activity, propagation, and survival of fungi responsible for food spoilage and mycotoxin production [23].

Generally, a_w_ is the main environmental parameter guiding food stability or spoilage. Knowledge of a_w_ enables prediction of food shelf life and potential fungal spoilage [22]. Based on this knowledge, the product a_w_ could be adjusted to improve their stability and safety. Fungal spoilage and mycotoxin production occur when the substrate a_w_ is adequate for the multiplication of the organisms involved [23].

The mean a_w_ values of corn grain, corn meal and grits (Table 1) from the 2015 crop differed significantly from the 2016 crop (*p* < 0.05).

Marín et al. [20] investigated the effect of a_w_ (0.89–0.97) and temperature (7–37 °C) on FB_1_ production by *F. verticillioides* and reported no FB_1_ production at 0.89–0.91 a_w_ regardless of temperature. Moreover, Mogensen et al. [21] reported that the optimal a_w_ value for fumonisin production by *Fusarium* sp. was 0.995 a_w_. Controlling a_w_ is one of the strategies to prevent fumonisin production in the post-harvest period and maintain safe storage of corn and derived products. Although some data presented a significant difference, all the mean a_w_ values (Table 1) were below 0.70, which is the critical a_w_ for safe storage [24].

The water activity of different milling fractions would influence the mycotoxin content, therefore, during industrial processing it is essential to reduce the available water to <0.70 a_w_ which is safe for storage.

The low a_w_ values in all the samples before and after the dry-milling process indicated good manufacturing practice because the industrial processing includes a wet degermination step that can increase a_w_ values. In addition, there was no significant correlation (*p* > 0.05) between a_w_ values and fumonisin levels in corn grain and milled fractions by the Pearson correlation test (data not shown).

The FB_1_ and FB_2_ levels in corn grain, endosperm, cornmeal, and grits (n = 320) from 2015 and 2016 are shown in Table 1. The most contaminated sample was corn grain, and FB_1_ was detected in 100% corn samples from both years with mean levels of 480 ng g^−1^ (2015) and 1080 ng g^−1^ (2016). Mean FB_1_ levels in corn grain were significantly different (*p* < 0.05) from other corn fractions (endosperm, cornmeal and grits) in both crops. Mean total fumonisin (FB_1_ + FB_2_) levels in corn grain samples from 2016 were significantly higher than those from 2015 (*p* < 0.05). Previous studies have shown high fumonisin frequency in corn and derived-products from Brazil [19,25,26]. Oliveira et al. [25] detected FB_1_ and FB_2_ in 100% of corn samples from Paraná State (n = 148) and the mean fumonisin (FB_1_ + FB_2_) levels were 3153 ng g^−1^, ranging from 63.8 to 66272 ng g^−1^. Ono et al. [26] analyzed 870 freshly harvested corn samples obtained from two points of the corn production chain and FB_1_ was reported in 100% of the samples, while FB_2_ was detected in 57 to 73.7% of the samples. Fumonisin levels (FB_1_ + FB_2_) ranged from 20.0 to 18780 ng g^−1^ (mean 1460 to 2870 ng g^−1^) [26]. Martins et al. [19] reported that mean fumonisin (FB_1_ + FB_2_) levels were 297 and 129 ng g^−1^ in cornmeal (*n* = 29) and corn grits (*n* = 28), respectively, while they were 311 and 206 ng g^−1^ in corn flakes (*n* = 11) and corn flour (*n* = 15), respectively.

**Figure 1:**
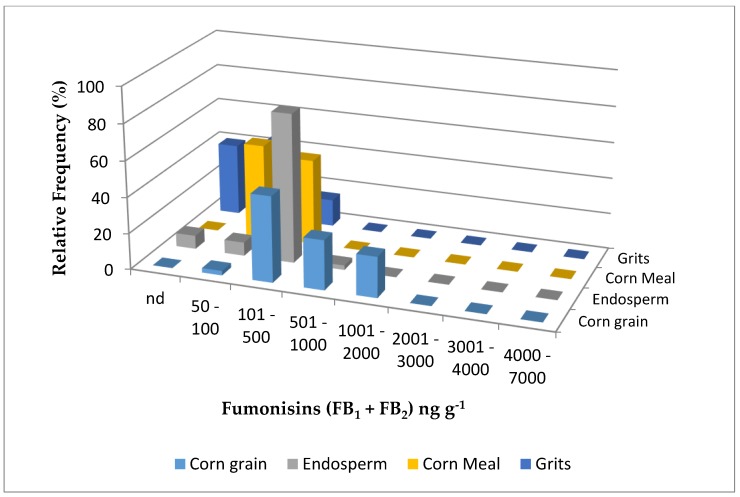
Distribution of fumonisin (B_1_ + B_2_) levels in corn grain, endosperm, cornmeal, and corn grits from two lots collected in a Brazilian corn milling plant in 2015. nd = not detected.

**Figure 2:**
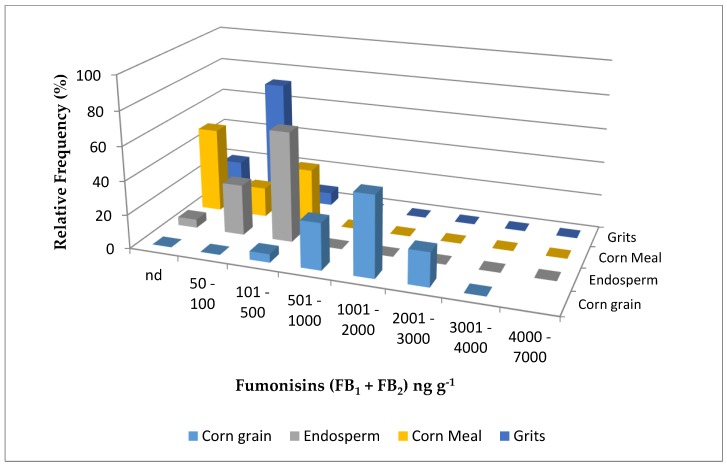
Distribution of fumonisin (B_1_ + B_2_) levels in corn grain, endosperm, cornmeal, and corn grits from two lots collected in a Brazilian corn milling plant in 2016. nd = not detected.

Figure 1 and Figure 2 show the fumonisin (FB_1_ + FB_2_) level distribution in corn grain and its derivatives from 2015 and 2016, respectively. All the positive corn grain samples from both years showed lower levels of contamination with fumonisins (B_1_ + B_2_) than the maximum levels allowed by the European Commission [27] and Brazil [28] (4000 ng g^−1^ and 5000 ng g^−1^, respectively) in unprocessed corn. Taking into account that the maximum level allowed for fumonisins in corn-based products intended for direct human consumption is 1000 ng g^−1^ [27], all the endosperm, cornmeal, and corn grits samples from both years were safe for human consumption, regarding fumonisins.

The low fumonisin contamination in endosperm, cornmeal and grits was due to the industrial processing that removed the outer parts of the corn grain (germ and pericarp) to produce corn-based products intended for human consumption from the corn endosperm. It has been reported that germ and pericarp are the corn fractions most contaminated by mycotoxins [29,30,31].

Dietary exposure degree is an important parameter for risk assessment and was expressed as fumonisin PDI. The PDI estimation is essential data for comparison with the health-based guidance value. Risk to human health may exist [13,32] when the exposure degree exceeds this value. The PDI was estimated using the data of household per capita food purchase of corn grain and corn-based products in Brazil [33] (Table 2), and the mean fumonisin levels of four corn lots and derivative fractions intended for human consumption collected from 2015 and 2016, considering 70 kg as average individual body weight. Concerning mycotoxin risk assessment, the dietary exposure evaluation is difficult when the concentration data are below the limit of detection (LOD), because in this case there is insufficient credibility in the quantitative results. The European Food Safety Authority (EFSA) has used substitution methods in the case of the non-detects data [34] according to the recommendations from the IPCS/GEMS [35]. Therefore, in this study the substitution method was used for handling non-detected data.

The corn grain was the sample that most contributed to fumonisin intake by the Brazilian population (85.0 ng kg^−1^ b.w. day^−1^) representing 4.3% of the PMTDI (2000 ng kg^−1^ b.w. day^−1^) (Table 2). Although cornmeal is the most consumed corn-based product (6.63 g/person/day), the PDI for fumonisins was only 10.3 ng kg^−1^ b.w. day^−1^ due to the low fumonisin contamination (107 ng g^−1^) (Table 2). Considering the intake of corn grain and corn-based products, the total PDI was 96.9 ng kg^−1^ b.w. day^−1^, which was lower than the fumonisin PDI for the Brazilian population reported by Caldas and Silva [18] and Martins et al. [19], but higher than those reported by Bordin et al. [17]. Caldas and Silva [18] estimated the total fumonisin daily intake for the total population in the Federal District and in the Brazilian population through the contamination of corn-based products obtained at local retail stores. The total fumonisin daily intake (FB_1_ + FB_2_) was 9.0% and 24.1% of the PMTDI in the Federal District and Brazil, respectively. Martins et al. [19] reported low fumonisin exposure (120 ng kg^−1^ b.w. day^−1^, 6.0% of the PMTDI) in the Brazilian population using fumonisin levels in corn-based products from markets in Paraná State, Brazil and the corn intake data from IBGE [33]. In Pirassununga, São Paulo State, Brazil, the PDI was estimated at 60 ng kg^−1^ b.w. day^−1^ (3.0% of the PMTDI) based on FB_1_ contamination of 120 corn-based products collected from 39 residences [17].

Based on the fumonisin levels in corn and its derivatives in this study, the fumonisin PDI was estimated for countries that import Brazilian corn, i.e., Europe, Malaysia, and Angola (Table 2). In European countries, the fumonisin PDI ranged from 35.7 ng kg^−1^ b.w. day^−1^ (France) to 177 ng kg^−1^ b.w. day^−1^ (United Kingdom), which was lower than the PMTDI of 2000 ng kg^−1^ b.w. day^−1^. The European Commission estimated the dietary exposure to fumonisins for the population of the European Union member states and it ranged from 5 ng kg^−1^ b.w. day^−1^ (in Norway) to 350 ng kg^−1^ b.w. day^−1^ (among Italian consumers) [12]. In Malaysia and Angola, corn and corn product consumption is higher than in the other countries evaluated (43.3 and 106 g/person/day, respectively) [36] and there is little information about the consumption of endosperm, cornmeal, and grits products, which was a limitation for estimating human exposure to fumonisins through intake of these corn fractions. Considering the fumonisin levels in corn grain, the fumonisin PDI in the Malayan and Angolan populations would be 634 and 1553 ng kg^−1^ b.w. day^−1^, respectively, which represent 31.7% and 77.6% of the PMTDI. However, considering the low fumonisin levels in corn derivatives (endosperm, cornmeal and grits), the fumonisin PDI would be between 70 and 290 ng kg^−1^ b.w. day^−1^ in these locations, assuming that these population groups only consume corn-derived products processed in Brazil. Corn consumption in Africa is one of the largest in the world and contributes to the high fumonisin exposure. In Transkei (South Africa), which has presented a high incidence of esophageal cancer, the PDI for fumonisins was 8670 ng kg^−1^ b.w. day^−1^, which was higher than the region with low esophageal cancer incidence (3430 ng kg^−1^ b.w. day^−1^), where corn consumption was lower [37].

Taking into account the low fumonisin levels in corn derivatives obtained by industrial corn processing, dietary exposure to fumonisins in populations in Brazil and importing countries was low, even for regions with high corn consumption. Therefore, all the samples could be considered safe for human consumption concerning fumonisins. Nevertheless, a continuous monitoring of corn contamination and the PDI evaluation are essential to estimate the human health risk. In addition, strategies to reduce exposure to fumonisins should be considered by corn processing industries.

## 3. Material and Methods

### 3.1. Sampling

The samples (corn grain, endosperm, cornmeal, and corn grits) were collected from one of the major Brazilian corn milling plants which receives samples from different farms. The plant is located in the North of Paraná State and processes about 12,000 tons of corn per month. In addition to internal consumption, it exports to Europe, Asia, and Africa.

Four lots (two from the 2015 and two from the 2016 crops) of freshly-harvested corn grain samples and their milled fractions, i.e., endosperm, cornmeal, and grits, were analyzed. The dry milling process of one lot corresponds to the processing of 500 tons of corn in 24 h. For each year, 160 samples were collected of corn grain (*n* = 40) and its fractions after the dry-milling process of products destined for human consumption, i.e., endosperm (*n* = 40), cornmeal (*n* = 40) and grits (*n* = 40), totaling 320 samples in two years. Dynamic sampling was carried out by collecting 10 kg of each sample, which were homogenized and about 500 g were removed, so that the milling fractions represented the lot of origin (whole corn grain samples). This procedure was repeated twenty times at five minutes intervals. All the samples were ground to pass through a 50 mesh sieve and then homogenized. Subsamples of 200 g were stored at −20 °C for FB_1_ and FB_2_ determination. In this plant, the endosperm is intended for exportation and used to produce breakfast cereals or cornmeal and grits. The cornmeal is used to prepare common dishes in Brazil and grits for corn flakes and snacks production.

### 3.2. Fumonisin Determination

FB_1_ and FB_2_ were determined according to the methodology of Shephard et al. [38] with some modification [39]. Ten grans of each sample (corn grain and its milled fractions) were extracted with 30 mL (methanol: water, 3: 1, *v/v*) by shaking at 150 rpm for 30 min followed by filtration. The cleanup step was performed using SepPak Accell plus QMA cartridges (Waters, Milford, MA, USA). After conditioning the cartridge, the filtered crude extract (1.0 mL) was applied and the cartridge was washed with methanol: water (3: 1, *v/v*, 6 mL) followed by methanol (J.T. Baker, Phillipsburg, NJ, USA; 3 mL). The fumonisins were eluted with 10 mL methanol: acetic acid (99.5:0.5) and evaporated to dryness under a stream of nitrogen at 45 °C. The analytical procedure involved sample derivatization with 200 μL O-phthaldialdehyde (OPA; Sigma, St. Louis, MO, USA) reagent. Twenty μL were injected into a reversed-phase isocratic HPLC system (Shimadzu LC-10 AD pump and RF-10A XL fluorescence detector) at excitation and emission wavelengths of 335 nm and 450 nm, respectively, using a C-18 Luna Phenomenex column (250 × 4.6 mm, 5 µm, Scharlau, Barcelona, Spain). The mobile phase was methanol: 0.1 mol/L NaH_2_PO_4_ (J.T. Baker; 80:20, *v/v*) adjusted to pH 3.3 with H_3_PO_4_ (J.T. Baker) at 1 mL/min flow rate. The detection (LOD) and quantification limits (LOQ) were determined as previously described [40]. The LOD for FB_1_ and FB_2_ were 27.5 and 35.3 ng g^−1^, respectively, and the LOQ for FB_1_ and FB_2_ were 45.8 and 58.8 ng g^−1^, respectively. Recovery rates were evaluated by spiking FB_1_ (100–1000 ng g^−1^ range) and FB_2_ (150–800 ng g^−1^ range) in the corn grain and endosperm. Recoveries for FB_1_ averaged 103.4% and 92.6% (mean CV 12.4% and 12.7%) and for FB_2_ 108.0% and 94.6% (mean CV 16.8% and 18.8%), respectively, based on triplicate analyses.

### 3.3. Food Consumption Data

The corn grain, endosperm, cornmeal and grits consumption data were obtained from a Household Budget Survey (HBS) conducted by the Brazilian Institute of Geography and Statistics (IBGE) [33] from May 2008 to May 2009. As the HBS does not specify the consumption of endosperm and grits, the sum of the consumption of foods derived from these corn fractions was considered. For endosperm, the data from corn flour and breakfast cereal were considered while for grits, the corn flakes data intake was considered. National data were collected from the food purchases of 55,970 households during seven consecutive days in urban and rural areas of all the Brazilian States. The food consumption data of each household was divided by seven and then by the household size to generate daily per capita consumption [33].

Taking into account that corn and its derivatives are exported to European countries, Malaysia and Angola, the PDI was calculated in these locations using the fumonisin contamination obtained by this study and the corn consumption data according to SCOOP [12] and FAO [36], assuming that these population groups only consume corn-based products processed in Brazil.

### 3.4. Estimation of Fumonisin Probable Daily Intake

The PDI was calculated using mean fumonisin (FB_1_ + FB_2_) levels (ng g^−1^) in corn grain and their derivatives of the four lots multiplied by the average daily consumption of each product (g/person/day) and divided by the average body weight (70 kg) [33], according to the formula:

$$\mathrm{PDI}\left({\mathrm{ng}\;\mathrm{kg}}^{-1}b.w.{\mathrm{day}}^{-1}\right)=\frac{\mathrm{food}\;\mathrm{consumption}\times \mathrm{mean}\;\mathrm{fumonisin}\;\mathrm{level}}{\mathrm{Average}\;\mathrm{body}\;\mathrm{weight}}$$

In the present study, fumonisin contamination of corn grain samples and their derivatives (endosperm, cornmeal and grits) was evaluated and the substitution method was used to handle non-detections, as recommended by the IPCS/GEMS [35] criteria adopted to estimate mycotoxin contamination when values less than the LOD were observed to avoid underestimating fumonisin exposure. The criteria were as follows: first, when all observations were over the LOD then the true mean were calculated; second, when the proportion of observations less than LOD was lower than or equal to 60%, the mean was calculated replacing those observations by ½ LOD. Third, when the proportion of results lower than the LOD was over 60% but lower than or equal to 80%, the mean was calculated replacing first those observations by 0 (zero) and second replacing them with the LOD. In the present study, less than 60% of the grits samples showed FB_1_ levels lower than LOD, and the mean was calculated replacing these observations by ½ LOD. Over 60% and less than 80% of the cornmeal samples showed FB_1_ levels lower than LOD and the mean was calculated replacing those observations first by 0 (zero) and then by LOD. Corn and endosperm showed contamination levels higher than LOD in more than 80% of the samples and the true mean was used to calculate the PDI.

### 3.5. Statistical Analysis

The normal distribution of the data was evaluated by the Shapiro-Wilk test in PAST software, version 3.22 (Hammer & Harper, Oslo, Norway). Differences in the mean water activity between corn grain samples and the milling fractions (endosperm, cornmeal, and grits) from 2015 and 2016 were statistically evaluated using the Student’s *t* test. Differences in the mean water activity between corn grain samples and the milling fractions (endosperm, corn meal and grits) from the same year were analyzed using ANOVA followed by the Tukey test (*p* < 0.05). The mean FB levels do not follow a normal distribution, therefore, non-parametric tests were applied. Differences in the mean fumonisin levels between corn grain samples and the milling fractions (endosperm, cornmeal, and grits) from the same year were evaluated by the Kruskal-Wallis test (*p* < 0.05). Differences in the mean fumonisin levels between corn grain samples, endosperm, cornmeal, and grits from 2015 and 2016 were statistically evaluated using the Mann-Whitney U test (*p* < 0.05). Statistical analyses were performed using the Statistica software, version 7.0 (Stat Soft, Inc., Tulsa, OK, USA).

## Figures and Tables

**Table 1:** Water activity, fumonisin B_1_, fumonisin B_2_, and total fumonisin (FB_1_ + FB_2_) levels in corn grain, endosperm, cornmeal, and corn grits from four lots (two from the 2015 and two from the 2016 crops) collected in a Brazilian corn milling plant.

Year	Sample	N	Water Activity (a_w_)	Fumonisin B_1_			Fumonisin B_2_			Fumonisin B_1_ + B_2_	
			Mean ^x^	Positive Samples (%)	Mean ^y^ (ng g^−1^)	Range (ng g^−1^)	Positive Samples (%)	Mean ^y^ (ng g^−1^)	Range (ng g^−1^)	Mean ^y^ (ng g^−1^)	Range (ng g^−1^)
2015											
	Corn grain	40	0.62 ^abB^	100	480 ^aB^	121–1441	53	283 ^aB^	142–568	631 ^aB^	121–1821
	Endosperm	40	0.64 ^aA^	93	198 ^bA^	95.0–569	-	-	-	198 ^bA^	95.0–569
	Cornmeal	40	0.61 ^bB^	100	118 ^cB^	41.0–287	5	53 ^b^	50.0–55.0	121 ^cA^	41.0–389
	Corn grits	40	0.60 ^bB^	60	86 ^cA^	36.0–208	-	-	-	86 ^cA^	36.0–208
2016											
	Corn grain	40	0.65 ^aA^	100	1080 ^aA^	303–2144	88	385 ^A^	77.0–785	1417 ^aA^	303–2863
	Endosperm	40	0.65 ^aA^	95	182 ^bA^	125–395	-	-	-	182 ^bA^	125–395
	Cornmeal	40	0.66 ^aA^	50	160 ^bA^	24.0–389	-	-	-	160 ^bA^	24.0–389
	Corn grits	40	0.65 ^aA^	80	54.0 ^cB^	22.0–256	-	-	-	54.0 ^cB^	22.0–256
2015 and											
2016 ^z^	Corn grain	80	0.63 ^a^	100	783 ^a^	155–2140	70	346	77.0–785	1026 ^a^	121–2730
	Endosperm	80	0.64 ^a^	94	191 ^b^	54.0–569	-	-	-	191 ^b^	54.0–569
	Cornmeal	80	0.63 ^a^	75	137 ^b^	41.0–389	2.5	53	50.0–55.0	137 ^b^	41.0–389
	Corn grits	80	0.63 ^a^	70	70.6 ^c^	36.0–256	-	-	-	70.6 ^c^	36.0–256

^x^ a_w_: the means between corn grain, endosperm, cornmeal and corn grits followed by different lowercase letters (a–b) or between the same type of sample, but in different years followed by different uppercase letters (A–B) are significantly different (*p* < 0.05) by the Tukey test and the Student’s *t* test, respectively. ^y^ FB_1_, FB_2_ and FBs: the means between corn grain, endosperm, cornmeal and corn grits followed by different lowercase letters (a–c) or between the same type of sample, but in different years followed by different uppercase letters (A–B) are significantly different (*p* < 0.05) by the Kruskal-Wallis test and Mann-Whitney test, respectively. z Average for 2015 plus 2016. Detection limit: FB_1_ = 27.5 ng g^−1^; FB_2_ = 35.3 ng g^−1^.

**Table 2:** Mean fumonisin (FB_1_ + FB_2_) levels in corn and derived products, average consumption, and probable daily intake in Brazil and in countries that import Brazilian corn.

Sample Type	Mean Fumonisin Levels (ng g^−1^)	Average Consumption (g/person/day)								Probably Daily Intake (ng kg^−1^ b.w. day^−1^)							
		Brazil	Europe					Malaysia	Angola	Brazil	Europe					Malaysia	Angola
			United Kingdom	France	Germany	Netherlands	Italy				United Kingdom	France	Germany	Netherlands	Italy		
Corn	1026	5.80	10.4	2.44	7.48	3.0	11.5	43.3	106	85.0	152	35.7	109	43.9	168	634	1553
Endosperm	191	0.59	1.20		18.4		1.50			1.62	3.29		50.4		4.11		
Cornmeal	99.9 ^a1^	6.63	14.9		3.99		1.00			9.46 ^b1^	21.2 ^b1^		5.69 ^b1^		1.42 ^b1^		
	107 ^a2^									10.3 ^b2^	22.7 ^b2^		6.09 ^b2^		1.52 ^b2^		
Grits	47.1	0.02			11.6					0.01			7.80				
Total		13.0								96.9 ^c^	177 ^c^	35.7	173 ^c^	43.9	173 ^c^	634	1553

Mean fumonisin (FB_1_ + FB_2_) levels were calculated according to IPCS/GEMS (1995) criteria [35]. a1 = first mean fumonisin (FB_1_ + FB_2_) levels calculated replacing non-detected samples by 0, according to IPCS/GEMS (1995) criteria when the results lower than the LOD is over 60% and lower than or equal to 80%. a2 = second mean fumonisin (FB_1_ + FB_2_) levels calculated replacing non-detected samples by LOD, according to IPCS/GEMS [35] criteria when the results lower than the LOD is over 60% and lower than or equal to 80%. Average consumption of corn and derived products in Brazil according to the Household Budget Survey (HBS) conducted by the Brazilian Institute of Geography and Statistics [33]. Average consumption in European countries based on data from the EU SCOOP Task [12]. Average consumption in Malaysia and Angola according to FAO Food Supply [36]. b1 = probably daily intake considering the first mean fumonisin level (FB_1_ + FB_2_) (a1). b2 = probably daily intake considering the second mean fumonisin level (FB_1_ + FB_2_) (a2). c = total probably daily intake considering the highest mean fumonisin level for cornmeal (FB_1_ + FB_2_) (a2).
